# Survey on Methods for Investigating Protein Functionality and Related Molecular Characteristics

**DOI:** 10.3390/foods10112848

**Published:** 2021-11-18

**Authors:** Yuqi Zhang, Siddharth Sharan, Åsmund Rinnan, Vibeke Orlien

**Affiliations:** 1 Department of Food Science, Faculty of Science, University of Copenhagen, Rolighedsvej 26, 1958 Frederiksberg C, Denmark or yuqi.zhang2021@gmail.com (Y.Z.); aar@food.ku.dk (Å.R.); 2 Paris-Saclay Food and Bioproduct Engineering Research Unit (UMR SayFood), Université Paris-Saclay, INRAE, AgroParisTech, 91300 Massy, France; siddharth.sharan@agroparistech.fr

**Keywords:** proteins, functional properties, molecular characterization, protein structure, spectroscopy, toolbox

## Abstract

Proteins from various sources are widely used in the food industry due to their unique functional performances in food products. The functional properties of proteins are somehow dictated by their molecular characteristics, but the exact relationship is not fully understood. This review gives a tangible overview of the methods currently available for determining protein functionality and related molecular characteristics in order to support further research on protein ingredients. The measurements of protein functionality include solubility, water holding capacity, oil holding capacity, emulsion property, foam property, and gelation. This review also provides a description of different methods of molecular characteristics including electrophoresis, surface hydrophobicity and charge, molecular interaction, and thermal property measurement. Additionally, we have put significant emphasis on spectroscopic methods (ultraviolet-visible, Fourier transform infrared, Raman, circular dichroism, fluorescence and nuclear magnetic resonance). In conclusion, first and foremost, there is a need to agree on a standardization of the analytical methods for assessing functional properties. Moreover, it is mandatory to couple different analyses of molecular characteristics to measure and monitor the structural changes obtained by different processing methods in order to gain knowledge about the relationship with functionality. Ideally, a toolbox of protein analytical methods to measure molecular characteristics and functionality should be established to be used in a strategic design of protein ingredients.

## 1. Introduction

The configuration of the protein molecule is complex, and many types of protein structures are found in biological materials. Overall, proteins are of animal or plant origins, with a huge diversity in their molecular characteristics and functions [1,2,3]. Animal globular proteins are found in blood and tissue fluids, while the fibrous proteins are found in muscle and connective tissue as well as hair and feathers. Plant proteins are typically classified according to the Osborne fractionation of seed storage proteins as albumins, globulins, prolamins and glutelins [3]. Proteins are the key component when structuring texture during food product processing. Different proteins have different functional performance because their specific characteristic and structural features varies, hence different proteins should be used for different food applications. Thus, protein’s central functional role underlines the need for a thorough understanding of how functionality is influenced and how it can be modulated and fine-tuned [4].

It is well-established that changes in the three-dimensional conformation of proteins are accompanied with a modification of the functional properties [5,6,7,8]. The techno-functional (non-nutritive) properties depend on the behavior of the protein molecule in a solvent or a particular food matrix upon various processing techniques. In relation to structural performance, protein functionality includes solubility, foaming, emulsification, and gelation, as well as water and oil holding capacity.

This communication surveys current common methods for investigating protein functionality and molecular structure aimed at addressing methodologies and facilitating future investigations about the interrelationship between them.

## 2. Protein Functionality Methods

Proteins have a vital role to play during preparation, processing, storage and consumption of foods due to the essential contribution to the quality and sensory features of the product [9]. Protein functionality is the central role that proteins play in a food matrix, including how they interact with other proteins, carbohydrates, and lipids together with small molecules like gases, salts, and water [3,10]. The functional properties of proteins can represent the functional behavior of single protein/ingredients in solution/dispersion including solubility, gel forming and rheological behaviors, emulsifying capabilities, foaming abilities and water and lipid binding capacities. The particular property depends on the specific molecular characteristics like size, primary, secondary, tertiary and quaternary conformations, charge distribution, as well as content and location of hydrophobic areas. These physicochemical properties are determined by both the environmental condition of the food matrix and the process operating steps.

### 2.1. Solubility

Protein solubility is of paramount importance for any protein to perform its functional role, which is a prerequisite for other functional properties including emulsification, foamability, and gelation [11]. Solubility is based on the protein–protein and protein–solvent interactions. Proteins can hydrate in solvents and thus conform into a thermodynamic low energy state of buried hydrophobic and exposed hydrophilic residues. A shift in these interactions can lead to folding/unfolding, and thereby decrease their ability to sustain themselves in the hydrated system. Solubility is thus a good indicator of the state of proteins in a particular environment [12].

Basically, protein solubility is a measure of how many protein molecules can stay dissolved in a given medium. Since proteins are often available as powders, the commonly used procedures of solubility tests are dispersing the protein powder (typically 1–3% *w*/*w*) in an aqueous or buffered solution, varying the pH with acid or alkali (typically 3–8), leaving it for a given time for hydration (short time stirring at ambient temperature or stored overnight at cold temperature) and measuring the protein concentration in the liquid phase (supernatant obtained by different centrifugation conditions). Usually, the protein solubility is calculated as [13]:

(1) $$\mathrm{Protein}\;\mathrm{solubility}\;(\%)=\frac{\mathrm{Protein}\;\mathrm{content}\;\mathrm{in}\;\mathrm{supernatant}}{\mathrm{Total}\;\mathrm{protein}\;\mathrm{content}}\times 100$$

Among solubility tests found in the literature, the solvent varies between water and different buffer systems. The simple protein-water interaction dynamics upon using water as solvent provides information of the pure protein solubility. Useful information regarding usage of the protein in food applications can be obtained by simulating food conditions with appropriate buffer capacity and ionic strength. Salt ions in the test system will shield or screen the electrostatic interactions between proteins and, thereby, affect the solubility through the salting-in or salting-out phenomena. Overall, the experimental results are largely affected by the actual testing conditions, such as the protein concentration used, buffer ionic strength, hydration conditions, and centrifugal parameters. Moreover, the method for measuring the protein concentration may affect the results [14].

The solubility is also related to the composition and sequence of amino acids in the protein chain (protein primary structure), as the amino acid may be hydrophobic, hydrophilic/uncharged or charged [11,15]. A protein with many polar amino acids on the surface possesses good soluble property in polar solvents. Typically, plant proteins are more soluble at low and high pH conditions and almost completely insoluble at their pI (pH 4–5), resulting in a U-shaped solubility pH-profile. For example, the alkaline soluble fraction extracted from pea protein isolate displayed a U-shaped curve with the lowest solubility at pH 5.0 and the best solubility at pH 8 [16]. Similar pH-solubility profiles were reported in the study of quinoa [17], soybean, and wheat protein [18].

### 2.2. Water Holding Capacity (WHC)

The WHC (sometimes also expressed as water binding or water absorption capacity (WBC or WAC)) refers to the ability to avoid water releasing from the protein’s three-dimensional structure [19]. WHC can also be considered as the ability of proteins to hold water when subjected to an external force (pressure, centrifugation, or heating) [20]. The interaction of proteins with water influence their other functional properties, such as solubility, foamability, emulsification, and gelation, but the knowledge on the nature of “bound” water and its relation to protein solubility is still superficial. In terms of dried powder products, such as protein concentrate or isolate, WHC is highly relevant and must be taken into consideration. First, when the protein powder is dispersed in water, water molecules are tightly bound to the surface polar sites of proteins by hydrogen bonding to form a “monolayer” of water. Second, further along in the progress of powder dissolution, water-water interactions are formed and multilayers of water are produced. The rate of water absorption of protein powders is related to the size of particles, the surface hydrophilicity, and the number of polar groups [20]. Overall, the WHC of a protein is affected by protein intrinsic properties and by concentration, ionic strength, pH, temperature, and process conditions. For example, salt ions in the solution will enhance water binding to protein, hence WHC increases at low salt concentrations [11]. In addition, other components of foods such as hydrophilic polysaccharides, lipids and salts also affect water binding properties.

**Figure 1:**
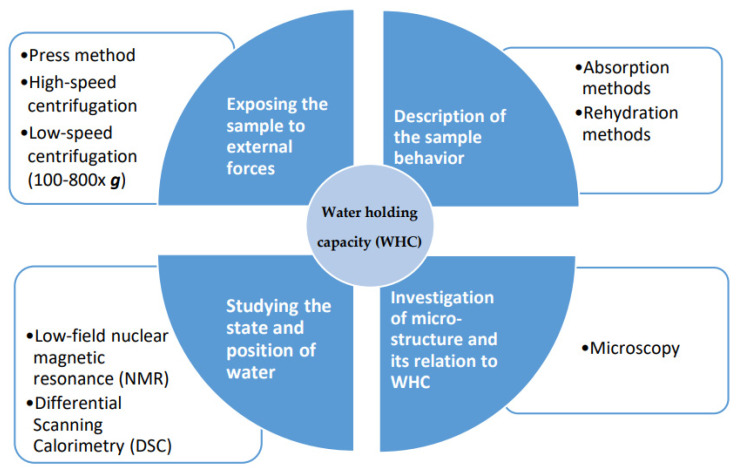
Various methods to measure water holding capacity (WHC). These can be divided into quantitative (the two upper) and qualitative methods (the two lower).

It is necessary to measure WHC to evaluate the interactions between protein and water in food systems. Over the past years, a large number of methods have been suggested to determine WHC of various food proteins. In general, the assessment of WHC is mainly divided into four types, as shown in Figure 1. Among them, a popular method is to assess the amount of moisture expelled when samples are subjected to external forces (e.g., pressing or centrifugation). Before testing, it should be noted that the parameters must be carefully selected to avoid massive structure damage during the measurement. When assessing dried protein powders, the procedure is to mix an exact amount of protein powder in an exact volume of water, followed by mixing and centrifuging the sample. After removing the supernatant, the weight of the hydrated pellet is recorded. The WHC is the amount of water retained by 1 g of protein powder and calculated as [2,21,22]:

(2) $$\mathrm{WHC}\;(\%)=\frac{\mathrm{Weight}\;\mathrm{of}\;\mathrm{water}\;\mathrm{held}\;\mathrm{by}\;\mathrm{sample}}{\mathrm{Weight}\;\mathrm{of}\;\mathrm{initial}\;\mathrm{sample}}\times 100$$

The WHC of proteins from different sources (broad bean, chickpea and lupin) was measured using the above method in the study conducted by Alu’datt et al. [23]. The results showed the highest WHC (44%) was observed for broad bean, while chickpea protein ranked second (42%), and lupin protein had the lowest WHC, at 33%. Bühler et al. [24] investigated the influence of dry-heat processing on the WHC of faba bean protein using soy protein as a control. The WHC did not change after dry-heating at 75 °C and 100 °C with the values of around 1.25 g g^−1^, while the WHC increased by 86.4% upon heating up to 150 °C. The effects of thermal processes on the WHC is also reported in isolated Kabuli chickpea proteins [25].

### 2.3. Oil Holding Capacity (OHC)

OHC also named oil or fat binding capacity (OBC or FBC), or oil or fat absorption capacity (OAC or FAC) is the ability of proteins to absorb and retain oil and to interact with lipid molecules, which is of great importance in food systems due to its effects on the distribution and emulsification of oil [26]. Compared with water binding, the mechanism of oil binding has been studied very little. Kinsella [27] indicated that oil absorption of proteins is primarily ascribed to the physical oil entrapment, which is determined by the protein microstructure. In addition, lipid molecules may interact with the non-polar side chains of the protein molecules [28,29]. Protein-lipid interactions are constituted by hydrophobic, electrostatic and hydrogen bonds. Hydrophobic bonds play a key role in the stability of protein-lipid complexes, which means that proteins with more hydrophobic regions display a better OHC. Oil absorption by food proteins is governed by processing conditions, the protein source, and concentration, as well as the type of oil and its distribution. For protein powders, particle size can also influence oil absorption; as the size of particles decreases, higher oil absorption capacity is, in general, observed [26].

Some of the methods used for WHC can also be applied for measuring oil holding, but not all are appropriate. Generally, OHC is determined by suspending an exact amount of protein powder in an exact aliquot of oil, mixing and centrifuging the sample, and decanting the remaining oil. The OHC is the ratio of the amount of oil retained over the mass of powder and calculated as [2,22]:

(3) $$\mathrm{OHC}\;(\%)=\frac{\mathrm{Weight}\;\mathrm{of}\;\mathrm{oil}\;\mathrm{held}\;\mathrm{by}\;\mathrm{sample}}{\mathrm{Weight}\;\mathrm{of}\;\mathrm{sample}}\times 100$$

This method was used to measure the OHC of six different pea cultivars in the study of Lam et al. [30], where there was no significant difference among them with values ranging from 3.1 to 3.3 g g^−1^. Similarly, the OAC of different proteins (wheat, soybean, rice, and pea protein) had the same OAC of around 1.2 g oil/g protein [18].

### 2.4. Foam Properties

A foam is defined as a two-phase mixture in which the gaseous phase is surrounded in a continuous phase (liquid or solid). However, foam is a thermodynamically unstable system, and will easily collapse unless stabilized. Some proteins are excellent foaming agents. Examples of protein-stabilized food foams are meringues, mousses, and ice cream. Generally, the process of protein foam formation is described in three stages. The first step is the diffusion or transport to the two-phase interface. After that, the structure of proteins is reorganized at the interface by protein unfolding with orientation of hydrophobic groups to the air phase. Finally, polypeptides interact with each other in the form of electrostatic, hydrophobic, hydrogen, and covalent bonds to form a continuous film. Hence, protein molecules can stabilize foams as a result of being adsorbed at the interface and serving as a cohesive monolayer around air bubbles. The solubility, surface flexibility and hydrophobicity of proteins are the main determinants of an efficient foam formation. Once the foam is formed, its stability is, in part, dependent upon the physical properties of the protein film. Protein-protein interactions and some environmental factors also determine the stability of foam. It is known that there are several ways of producing foams [31,32]. Foams can be generated using mechanical aeration [7,30,33,34,35,36,37] or by air sparging [38]. Mechanical aeration is more commonly performed either by mixing [33,34], whipping [35] or high-speed homogenization (e.g., ultra-turrax) [7,30,36].

**Figure 2:**
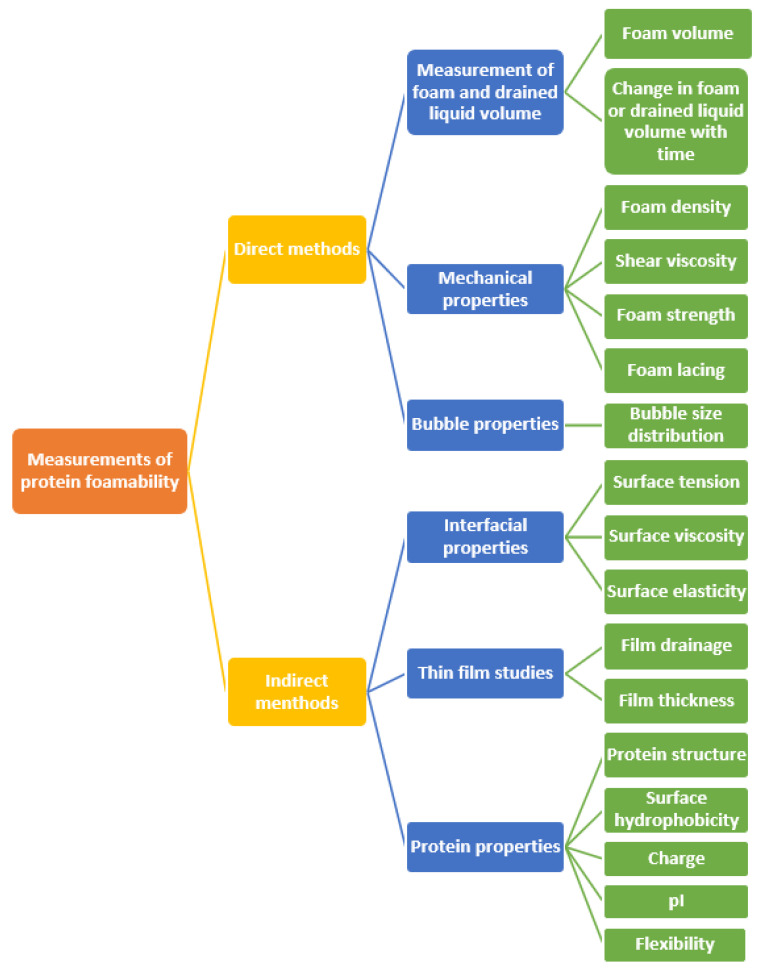
Common direct and indirect measurements of foam properties.

As shown in Figure 2, the assessments of protein’s foam properties are divided into direct and indirect methodologies. Direct methods give the physical information on the foam for comparison between different foams. In contrast, indirect measurements are those made on a model system at the microscopic and sometimes molecular level to explain foam behavior. In the actual testing conditions, the choice of method should be considered in relation to the objective of application and the protein system. Overall, the foam properties of proteins are mainly assessed by the foam capacity (FC) and foam stability (FS).

The most used method for assessing FC and FS, which is also most related to applications is the measure of foam volume. It has been applied for determining the foam property of different proteins such as wheat, soybean, rice [18], pea [22,30,39], and quinoa protein [17]. The procedure is to dissolve the protein in a specific volume of water or buffer (dependent on the application pH), homogenize the solution (by the method previously described) and measure the foam volume. The FC is typically calculated by [2,30]:

(4) $$\mathrm{FC}\;(\%)=\frac{V_{F}}{V_{L}}\times 100$$

where *V_L_* is the volume of the initial solution and *V_F_* is the volume of the formed foam. It is noted that various forms of this equation are found in the literature.

The foam stability is more commonly calculated as the rate of foam volume decrease or liquid drainage from the foam with time [2,30]:

(5) $$\mathrm{FS}\;(\%)=\frac{V_{30}}{V_{F}}\times 100$$

where *V_F_* is the foam volume immediately after production and *V*_30_ is the volume of the foam after 30 min storage.

### 2.5. Emulsion Properties

An emulsion contains two immiscible liquids, one which is dispersed (as droplets) within the other phase. The former is commonly called the dispersed, internal, or discontinuous phase, and the latter is the external or continuous phase. Food emulsions are normally made up of lipid and water, therefore, the two major types of emulsions are ‘oil-in-water (O/W)’ and ‘water-in-oil (W/O)’. The emulsion is inherently thermodynamically unstable owing to the interfacial tension existing in the interface between two phases. When the interfacial tension increases with an enhanced area of contact, the stability of the emulsion will be broken down more easily. Therefore, amphiphilic molecules or emulsifiers are used to help decrease the interfacial tension, thus slowing down separation and stabilizing emulsions [40].

Proteins are widely used as emulsifiers in food systems since they can migrate to the interface and orient their polar and non-polar amino acid residues towards the aqueous and lipid phase, respectively, thereby developing a stable coating around the droplet. Examples of protein-stabilized emulsions are mayonnaise (O/W), ice cream (O/W) and butter (W/O). The characteristics of a protein such as the solubility, ease of denaturation, isoelectric point, surface hydrophobicity and surface charge play key roles for its ability to emulsify. The emulsification ability of protein is also affected by a variety of factors such as temperature, pH, ionic strength, processing conditions and the viscosity of the aqueous phase. In general, an emulsion is made by mixing the two liquids, but can be produced either in a crude or fine manner. Crude or coarse emulsions can be produced by blenders or dispersion homogenizers, whereas fine emulsions are produced by pressure homogenizers [41].

The emulsion (or emulsifying) properties of a protein are assessed by the emulsion capacity and emulsion stability. However, various parameters are used to characterize them, such as emulsion capacity index (ECI), emulsion activity index (EAI), emulsion stability index (ESI), emulsion volume index (EVI), and creaming index (CI) [42]. Along with the various parameters, different methods and procedures have been applied for the determination of emulsion properties. A classic method of emulsion capacity is to determine the volume of oil emulsified by the unit weight of protein. In this method, oil is added until emulsion breaks down and the endpoint is indicated by a drastic reduction in conductivity [43]. Besides, the emulsion property can be evaluated by measuring the height of the emulsified layer. The emulsion capacity is expressed as [44]:

(6) $$\mathrm{EC}\;(\%)=\frac{H_{1}}{H_{0}}\times 100$$

where *H*_0_ is the total height of content, and *H*_1_ is the height of emulsified layer after centrifugation.

After that, the emulsion is heated at 80 °C for 30 min, followed by centrifugation. The emulsion stability is calculated by [44]:

(7) $$\mathrm{ES}\;(\%)\;=\;\frac{H_{2}}{H_{0}}\times 100$$

where *H*_2_ represents the height of remaining emulsified layer after the above treatment.

Another typical method for evaluating both capacity and stability is the measurement of oil droplet size distribution by laser light scattering equipment. The capacity of a protein to form an emulsion and keep it stable is dependent on its ability to form small droplets, since smaller oil droplet sizes are indicative of higher quality. Hence, the volumetric mean diameter (*d*_4,3_) is calculated from the particle size distribution as a function of volume immediately after emulsification (EC) and upon storage at given conditions and time (ES) [45,46]. The turbidimetric method is also widely used to determine the emulsifying properties. The emulsion at 0 and 10 min after homogenization was diluted with SDS solution followed by measuring the absorbance at 500 nm. The results were expressed as [18]:

(8) $$\mathrm{EAI}\;(m^{2}/g)\;=\;\frac{2\;\times \;2.303\;\times \;A_{0}\;\times \;N}{\varphi \;\times \;C\;\times \;10^{4}}$$

(9) $$\mathrm{ESI}\;(\min)\;=\;\frac{A_{10\;}\times \;10}{A_{0\;}-\;A_{10}}$$

where *A*_0_ represents the absorbance at 0 min after homogenization, *N* is the dilution factor, *C* is the protein concentration before emulsion formation (g/mL), *φ* is the oil volume fraction of the emulsion, *A*_10_ represents the absorbance at 10 min after homogenization.

### 2.6. Gelation

A protein gel is a three-dimensional cross-linked network of protein molecules. Protein gels may hold water, fat, sugars, and other constituents [11]. The process of gel formation is based on a (partial) protein denaturation with resultant conformational changes (access to active sites), followed by interactions, aggregation and final gelation [47]. It is noted that the well-ordered network is formed by a good balance of repulsive and attractive forces by hydrophobic and electrostatic interactions, as well as hydrogen and disulfide bonds [48,49]. Protein gels are formed either under heat-induced or cold-set gelation conditions. A temperature above the protein’s denaturation temperature is a prerequisite for the heat-induced gel formation. The cold-set gel can be induced by salts, acidifying agents, or enzymes, which is an alternative access to control the soluble protein aggregation [47]. For example, protein-based gels can be produced either by heat or pH [34,50]. Overall, the heat-induced gels take up the majority of food gels. Protein gelling properties are influenced by both intrinsic properties (protein concentration, amino acid composition, molecular weight, and hydrophobicity) and environmental factors (solvent parameters, heating conditions, pH and ionic strength).

Gel are characterized by: (i) rheological methods such as the least gelling concentration (LGC), storage modulus (G′), loss modulus (G″), tanδ (G″/G′), and gelation temperature; (ii) mechanical deformation properties including stored recoverable energy, fracture strain (gel brittleness), Young’s moduli (gel stiffness), fracture stress (gel strength), and elasticity [47]. LGC is probably the simplest method to perform, since it is determined by heating protein dispersions (with protein concentration in the range of 1–20% *w*/*v*) in a tube for one hour, cooling and checking gel-formation by inverting the tube. LGC is considered as the lowest concentration at which the sample does not flow after inversion [16]. Normally, the LGC value of pea protein ranged from 14% to 17% [47]. In general, the lower LGC, gelation temperature, G″ and tanδ-values, the better is the ability to form gels with elastic networks. High G′ values demonstrate that the protein gel has stronger networks with increased protein-protein interactions [47,51]. Likewise, good textural properties are normally expressed by high values of mechanical deformation parameters.

## 3. Molecular Characterization Methods

In this section, a group of molecular characterization methods are presented to understand physicochemical and structural properties associated with protein functionality.

### 3.1. Electrophoresis

Protein molecular weight distribution is characterized by electrophoresis. The migration of proteins in an electric field depends on their charge, molecular shape and mass [52]. The most commonly used method is sodium dodecyl sulfate-polyacrylamide gel electrophoresis (SDS-PAGE). Typically, the proteins are diluted in a SDS sample buffer (non-reducing condition) or with dithiothreitol (DTT, reducing condition), following loading on a gel together with a marker and electrophoresed. After separation, the proteins are fixed and stained with a colour agent. The proteins can then be identified by comparing relative mobilities to the molecular weight standards. It is emphasized that in the SDS–PAGE analysis, it is advised that the same total protein concentration is loaded on each lane, so the PAGE result shows the relative distribution of the individual solubilized proteins in a comparable mode [52]. Moreover, the band-size intensities can be analyzed by semi-quantitative comparison of their pixel intensities in the gel, thereby obtaining an evaluation of the degree of solubilization. The disappearing of bands or newly formed bands on the gel is attributed to either degradation of larger proteins into small sub-fragments or aggregation of low-molecular-weight proteins. For example, the SDS-PAGE profile of heated pea protein showed that the band >100 kDa are probably formed due to heating-induced aggregation [39]. It is emphasized that SDS-PAGE analysis does not reveal if the proteins are native or denatured.

### 3.2. Surface Hydrophobicity (So) and Surface Charge

Hydrophobicity is defined as the tendency of non-polar solutes to adhere to each other in aqueous conditions [53]. The crystallographic investigation of protein structure has found that there are some hydrophobic residues partly exposed on the molecular surface which play a key role in studying conformation and interactions of protein molecules. Furthermore, the number and size of hydrophobic amino acids are significantly related to protein solubility and states of aggregation [54]. Therefore, it is important to measure *So* to better understand protein functional properties. There are several ways to determine *So* [5,55]. The classic measurement of *So* is through probe spectrofluorometry using 1-anilinonaphthalene-8-sulfonic acid (ANS) or cis-parinaric acid (CPA). For example, the probe of ANS was used to investigate the effect of industry-scale microfluidization [56], high intensity ultrasound [57] or the glutaminase treatment [58] on *So*. This method is quick and easy to operate, but the results obtained may be influenced by the interaction between the probes and the proteins. In addition, the detergent binding method is proposed to quantitate the binding of a hydrophobic ligand to proteins as an assessment of *So*. In the study of Tang et al. [59], *So* was expressed as the amount of SDS bound to the protein. Hydrophobic-interaction chromatography and hydrophobic partition are another two commonly used methods to measure hydrophobicity.

The charges on the surface of protein molecules can be negative, neutral, or positive. The surface charge is usually evaluated by surface charge density and surface potential. The surface charge density describes the charge distribution on the surface, which is calculated as the net number of charges per unit surface area. The surface potential reflects the free energy required for the surface charge density changing [60]. In laboratories, the surface potential is generally reported as zeta-potential (ζ, mV). For example, Cui et al. [46] detected that the zeta-potential of pea proteins from different cultivars at an extraction pH of 9.0 were all in the range of around +30 mV to −30 mV. The zeta-potential is mainly measured by a micro-electrophoresis device. This instrument records the velocity and direction of the particle moving in an applied electrical field and calculates the electrophoretic mobility. After that, the electrophoretic mobility is converted into zeta-potential by dedicated software.

### 3.3. Thermal Property

Protein thermal denaturation helps understand their structure-functional potential. When proteins are subjected to changes in temperature (e.g., during processing), heat exchange (endothermic or exothermic) will occur due to various physical or chemical changes. A differential scanning calorimeter (DSC) has been extensively applied for determining the thermal physical transitions of proteins due to temperature. Specifically, conformational changes, like denaturation, of proteins upon heating (or cooling) can be observed [61,62]. The DSC thermogram describes changes in Gibbs free energy, enthalpy, and heat capacity during protein unfolding or denaturation [62]. In the transition from native to denatured protein states, energy is absorbed and enthalpy decreases. For example, Puppo et al. [63] observed that soybean protein isolates displayed a reduction of enthalpy in their denatured state. In addition, the differences of protein sources can be explained by thermal denaturation profiles. Oat protein denatures at 112 °C and soybean proteins denature at 93 °C, while field pea proteins denature at 86 °C [64]. The effects of different processing conditions such as phosphorylation, thermal processing, and high pressure on thermal properties of pulse proteins have also been explained from DSC thermograms [22,25,63].

### 3.4. Molecular Interactions

Proteins may interact with themselves (or other components) resulting in changes in their functional properties. During various protein extraction methods or food processing techniques, molecular changes may occur due to breaking or formation of chemical bonds and/or disruption or stabilization of non-covalent interactions. Hence, the new macroscopic structure appears due to the proteins forming protein-protein aggregates, which may lose functionality, often as insolubilized complexes. These changes are clearly complex, involving alteration of both covalent, e.g., inter- and intramolecular disulfide bonds, and non-covalent ones, such as hydrogen, electrostatic, ionic and hydrophobic, interactions. It is the relative proportion of each type of bond and interactions in the structural ensembles that determines their formation and change in functionality. For example, in some product structuring, the non-covalent bonds play a dominant role over disulfide bonds, while in others the non-covalent and disulfide bonds are both important. Therefore, to figure out the new protein conformation and related modification of their functional properties, differentiation and understanding of the specific protein–protein interactions is important. The most common approach of studying these interactions is protein resolubilization by selective reagents with known mechanisms of protein solubilization [65]. The method is based on the premise that proteins (and structural formations) can be solubilized with extracting solutions containing an agent capable of breaking chemical bonds and disrupting non-covalent interactions. For example, reducing agents like dithiothreitol (DTT) and 2-mercaptoethanol will break disulfide bonds, urea, thiourea and sodium dodecyl sulfate (SDS) will break non-covalent interactions. Urea is more efficient in breaking hydrogen bonds, while thiourea is generally used to break hydrophobic interaction. If the extraction solution contains a combination of all reagents in the study, it is known as the isoelectric focus (IEF) buffer. It is noted that upon characterizing and explaining the relative importance between disulfide bonds and non-covalent interactions, different conclusions can be made from the solubility results depending on the type of extracting systems and the baseline used for comparison [65]. Therefore, it is recommended to use the IEF buffer system with the omission of one or more selective reagents for a correct protein solubility study [65]. Thus, the following conditions should exist when investigating molecular interactions: (1) an IEF solution that contains all of the reagents to break all of the possible bonds, (2) other extracting solutions without one or more reagents from the IEF solution, and (3) the comparison of solubility values of other extractants with that of the IEF solution [65]. For instance, Chen et al. [66] investigated chemical bonds of soybean protein and their relative importance during extrusion cooking using the above procedure.

### 3.5. Spectroscopy

Spectroscopy is an experimental subject dealing with electromagnetic radiation-matter interactions. Electromagnetic radiation provides different kinds of interactions with the matter in a wide range, from radio wave to gamma rays, therefore developing various types of spectroscopic techniques [67]. These techniques are popular in the field of food and health sciences, and also in monitoring conformational changes of proteins, as they are fast, simple, non-destructive, convenient, and sensitive. Common spectroscopic techniques include ultraviolet-visible (UV-Vis), Fourier transform infrared (FTIR), Raman spectroscopy, circular dichroism (CD), fluorescence, and nuclear magnetic resonance (NMR) spectroscopy.

#### 3.5.1. Ultraviolet-Visible (UV-Vis) Spectroscopy

The measurement of UV–Vis spectroscopy is concerned with the electronic transition occurring within the range of 200–780 nm. The types of electronic transition include π−π* and *n*−π* transitions [67]. Several chemical molecules and specific chromophores can produce absorption spectra under UV-Vis light. The absorption in the area of 230–300 nm is predominantly due to side–chains of aromatic amino acids including phenylalanine (Phe), tyrosine (Tyr) and tryptophan (Trp) residues [68]. Overall, UV–Vis absorption spectroscopy is a commonly used tool to probe the conformational changes of proteins by detecting and monitoring the microenvironment of aromatic amino acid residues. For example, recent studies have reported the effects of microfluidization treatment and ultrasound on protein structural changes using UV–Vis spectroscopy [69,70]. The UV-Vis determination is relatively simple, and the results can usually be reproduced between different laboratories. However, compared with other spectroscopic techniques, UV–Vis spectroscopy is less selective, as different species may absorb UV light in the same region [71]. As an example, cysteine and disulfide bonds also exhibit rather low peaks (around 260 nm) [68,71], which results in the inability to measure which chromophores make changes through zero-order UV spectra. Therefore, second- or fourth-derivative spectra is used to visualize the minor changes of UV absorption peaks [8].

#### 3.5.2. Fourier Transform Infrared (FTIR) Spectroscopy

As a vibrational spectroscopic technique, infrared spectroscopy relies on vibrations of the atoms of a molecule in the region of 4000–400 cm^−1^, primarily stretching and bending motions [72]. In general, FTIR spectroscopy is employed to obtain protein secondary structural information. There are nine infrared absorption bands from proteins and peptides, referred to as A, B, and I–VII. Of these, the amide band I is regarded as the primary absorption region to diagnose protein secondary structures, which mostly arises from the C=O stretching vibrations near 1650 cm^−1^ [73,74]. Several components representing different secondary structures including α-helices, β-sheets, turns and random coils overlap with the amide band I, which makes it hard to differentiate them. Fourier self-deconvolution and second (and higher) derivatives are applied to narrow bandwidths for qualitative and quantitative analysis [74]. The measurement of FTIR spectroscopy is rapidly conducted with small sample volumes. In particular, FTIR spectra is restricted by environmental conditions. The determination of samples may be interfered with by the strong IR absorbance of H_2_O at 1640 cm^−1^, therefore overlapping the protein signal in this region [72,73,74]. However, to what degree this overlap influences the data analysis and interpretation of the IR-signal has yet to be detailed or described. It was reported that FTIR is used to evaluate the effects of different processing treatments, such as pH [46], low moisture extrusion [75], and phosphorylation [22], on secondary structure characterization.

#### 3.5.3. Raman Spectroscopy

Raman spectroscopy is a technique exploiting inelastic scattering of samples exposed to light radiation. During measurement, Raman scattering generally changes samples’ vibrational or rotational energy, leading to a wavelength shift (Raman shift) shown in the spectrum [76,77]. Raman spectroscopy specializes in detecting protein secondary structures according to specific Raman shifts of amide I and III bands corresponding to each secondary structural element. As with infrared spectroscopy, quantitative analysis can also be performed using Raman spectroscopic data. Apart from the characterization of amide bands, Raman spectroscopy can also be utilized for detecting the microenvironments of amino acid chains. For instance, the Raman vibrations at 833 and 860 cm^−1^ are ascribed to Tyr residues [78]. In the Raman spectrum, there is very little overlap between water molecules and other molecules. As a result, a significant advantage of Raman spectroscopy is that there is less interference of water molecules compared to IR, which allows the determination of water-rich samples, such as food matrices [76]. From this point of view, Raman spectroscopy is regarded as a complement to infrared spectroscopy. However, strong fluorescence background is a non-ignorable problem regarding Raman spectroscopy. The presence of fluorescence will cover some weak Raman signal values, in particular at lower Raman shifts. Some methods reduce the influence of fluorescence using lower energy light sources, time-domain methods, SERS that amplify the Raman signal, baseline correction, and the application of quenchers [79,80].

#### 3.5.4. Circular Dichroism (CD) Spectroscopy

CD spectroscopy is another tool to determine protein structure, protein folding property, or interactions of proteins with ligands. During practical testing, the plane polarized radiation will be divided into left and right circularly polarized components when passing through the samples in an alternating 50 kHz electric field. Unequal absorption between these two components will result in elliptical polarization. The measurement of CD is on a basis of the difference in absorbance, which can be expressed as ΔA = A_L_ − A_R_. The results can also be reported via ellipticity (θ) in degrees with the equation of θ = tan^−1^ (b/a) in which a and b are the major and minor axes of the ellipse [81,82]. Samples possessing optical activity can be detected by CD spectroscopy, such as amino acids. CD spectroscopy, therefore, is well suited to study protein secondary structure in the far UV region of 170–240 nm and the tertiary environment of aromatic amino acid residues in the near UV region of 260 to 300 nm. In the far UV region, the spectrum is related to π−π* and *n*−π* transitions of amide groups with the effect of the geometries of the polypeptide backbones. Each of the secondary structural elements tends to have a characteristic wavelength region [83]. While in a near UV region, the specific spectra arise from the absorption of specific aromatic amino acids. For example, Trp displays a clear peak in the area between 290 and 305 nm [81,84]. Generally, the analysis of CD spectra is performed with the help of some computational methods, such as neural networks, optimization algorithms, regression, or singular value decomposition [84]. Compared to other techniques such as NMR, CD measurement is conducted rapidly using small amounts of samples. Besides, CD spectra can be acquired in different environments, such as pH, temperature, and in solid states and solutions. But the usage of CD spectroscopy may be limited by its relatively low resolution [81,82,85].

#### 3.5.5. Fluorescence Spectroscopy

Fluorescence is described as a process in which molecules excited by the absorption of UV-Vis light return to their ground state through the emission of photons. The energy of emission is smaller than that absorbed, and the difference between them is named the Stokes shift [86,87]. The characterization of fluorescence is related to properties of the molecules and their microenvironment. Intrinsic fluorescence of buried and/or exposed protein residues indicate the folded state of protein tertiary conformation. Tryptophan, tyrosine and phenylalanine are intrinsically fluorescent residues with tryptophan residues being popularly studied for intrinsic fluorescence by virtue of its high extinction coefficient compared to the others [88,89]. Maximum fluorescence intensity (FI_max_) and its corresponding wavelength (λ_max_) are commonly used to characterize protein conformational changes. For example, Xiong et al. [57] found that the FI_max_ of ultrasonicated samples decreased and their λ_max_ shifted from 334.8 nm to 338.0 nm. Besides, fluorescence spectroscopy is a useful tool to determine protein hydrophobicity, as mentioned in Section 3.2. Typically, two conventional spectra (fluorescence emission and excitation spectra) are sufficient for a basic single-fluorophoric model system. However, real food samples are complex with multiple fluorophores, therefore it is recommended with more advanced techniques, including excitation-emission matrix (EEM) fluorescence spectroscopy, synchronous fluorescence spectroscopy (SFS), and total synchronous fluorescence spectroscopy (TSFS) [90,91]. Their data analysis is performed by chemometrics methods such as principal component analysis (PCA), the partial least squares (PLS) regression algorithm, linear discriminant analysis (LDA), and others [87]. Fluorescence works well when probing minor and trace components in complex samples, but its detection is restricted to the samples containing fluorescent components. Moreover, further investigations into the influence of factors such as chromophores, quenchers, pH and temperature on the signal itself is needed to better understand and handle these signal artefacts.

#### 3.5.6. Nuclear Magnetic Resonance (NMR) Spectroscopy

NMR provides specific structural, energy, and dynamic information of molecules by utilizing the magnetic properties of certain nuclei. ^1^H, ^13^C, ^15^Nand ^31^P are commonly used nuclei in food science. These elements are part of most compounds in food, meaning that there is at least one detectable nucleus, and NMR is therefore regarded as a universal detector. In terms of the sample with multiple types of nuclei, it is able to conduct different food analyses by choosing corresponding nuclei detection [92]. NMR signals are generally characterized by a series of parameters such as chemical shifts (δ), scalar coupling (*J* coupling), the peak intensity, longitudinal relaxation (T_1_), transverse relaxation (T_2_), and the nuclear Overhauser effect [93]. For example, the ^13^C NMR spectroscopy was used for investigating the molecular structure of soy protein because the chemical shift is associated with the main-chain conformations such as α-helix and β-sheet [94]. In addition, Kass and Craik [95] summarized various applications of NMR in the folding behavior, dynamics, structures, and interactions of plant proteins.

## 4. Relationship between Structural and Functionality Features

Protein properties are dependent on their intrinsic aspects (primary, secondary, tertiary, and quaternary structure) but also their extrinsic aspects (solvent, ionic strength, pH, etc.) to enable their functional potential. To take an example of proteins from plant sources, the different types of proteins present, including albumins, globulins, prolamins and glutelins, have different functional capacities owing to their differences in structure. For instance, albumins, which are water soluble, are of smaller size (5–80 kDa), whereas native globulins (180–360 kDa) are salt soluble proteins. However, it is the globulins that are mostly associated with functionalities [31,32]. Pulse globulins, including legumins, vicilins and convicilins, have differences in all degrees of conformations. Legumin chains are joined by disulfide bonds, whereas vicilins lack disulfide bridging [32]. Furthermore, legumin is a ~360 kDa hexamer with 20 kDa subunits, while vicilin is a ~180 kDa trimer with 50–60 kDa subunits [31,32,34]. Considering functional capabilities, 7S vicilins are associated with higher emulsifying capability and gel strength [33,36]. Legumins do not coagulate at 100 °C, whereas vicilin coagulates at 95–100 °C [35]. 11S fraction of globulins are associated with higher hardness, springiness and cohesiveness of curds along with greater foam and gelling capacity. While legumins aid in the expansion of foams and gels, vicilins help stabilize foams and emulsions [7]. Functional protein properties depend on its behavior in a given food matrix when exposed to the processing needed to achieve a particular product. These processing steps can cause structural changes that likely will affect the functionality of the protein. Exact changes in protein structure due to different conditions, leading to certain gain or loss in functionality, are yet to be explored.

Emulsion, foam and gel characteristics have been attributed to protein solubility, charge, viscosity, interfacial tension and viscoelasticity, which in turn are strongly connected to the protein structure. For example, protein transformations in association with functional properties of pulse proteins have been reported in pea [30,39] and lentil proteins [96]. Protein hydrophobicity detected by fluorescent probes has been associated with emulsifying properties [39] and with foaming properties [7,25,30]. Studies have been conducted for protein isolates, which are relatively pure proteins, while other studies are on flours or concentrates that are multicomponent in character [97]. Thus, their functional properties are likely dependent on the presence of other components. A recent study addresses the association of both protein and non-protein components with functionalities [31]. Overall, understanding the structure-functional relationship between proteins at food utilization conditions could give an idea of the functional potential of the proteins.

**Figure 3:**
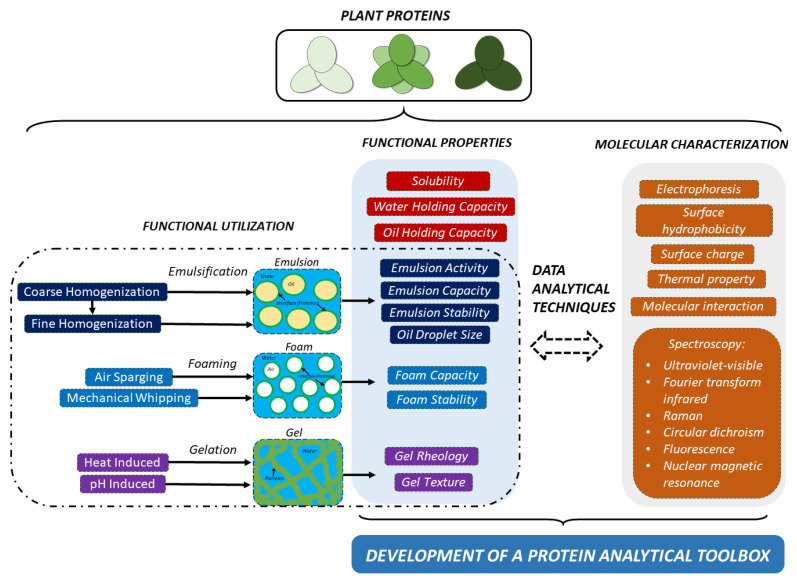
Illustration of functional utilization, functional properties, molecular characterization, and the protein analytical toolbox used to characterize proteins.

Some studies report experimental set-ups to investigate the relationships between structure and functionality [6,97,98,99]. As seen in Figure 3, in the research approach towards understanding of functionality and the related molecular characteristics, there are four basic elements: (1) functional utilization of the ingredient in concern, (2) functional evaluation of the ingredient using instrumental techniques explained in the sections above, (3) evaluation of the molecular characterization concerning proteins, and finally (4) using data analytical techniques to establish a screening protein toolbox, which is an assembling of methods and instrumentation for obtaining information on protein structure modification and strategies for further in-depth investigations of both structural and functional changes. For instance, a recent study showed the importance of using statistical analytical tools (PCA and Pearson’s correlation) for a large set of data obtained from fava bean concentrates processed by different process conditions [97]. Considering how the structure affects the functional role of proteins, it will be interesting to classify different proteins using the toolbox. We believe that it will be possible to apply this protein toolbox to the food matrix for efficient or improved functionality and the generation of new foods.

## 5. Conclusions

This survey has presented methods for measuring protein functionality and potential methods for evaluation of related molecular characteristics to support research on protein ingredient development. There has been a great amount of scientific and applied work done in utilizing different protein sources as new generations of functional ingredients. However, a strategy for testing functional properties and their relationships with physicochemical and structural characteristics in order to improve knowledge about proteins has yet to be developed. Variations in performing the analytical methods are found in different papers, impeding the possibility of compare results and deducing general findings. The protein functionality analyses presented are the ones most frequently used, and are an attempt to stimulate a standardization of the analytical method for assessing functional properties. It is anticipated that a protein analytical toolbox that couples different chemical, physical and spectroscopic analyses in relation to protein functionality and molecular characterization will improve the rational design of different protein ingredients. Such an important protein toolbox is currently being developed in our laboratory. An important area for further improvement includes methods for evaluating protein and non-protein interactions in relation to the impact on protein functionality, which is highly relevant for protein concentrates and flours. New research directions within spectroscopic techniques could connect the obtained data through data fusion among different spectroscopic techniques. For example, the low resolution of CD could be increased by data fusion with fluorescence spectroscopy or FTIR. Furthermore, more advanced analysis of NMR data would be beneficial for the extraction of high-quality information from complex systems, such as ingredients and foods.
